# Serum haptoglobin dynamics in pigs vaccinated or not vaccinated against porcine circovirus type 2

**DOI:** 10.1186/2055-5660-1-3

**Published:** 2015-04-16

**Authors:** Lorenzo Fraile, Yolanda Saco, Llorenç Grau-Roma, Miquel Nofrarías, Sergio López-Soria, Marina Sibila, Antonio Callén, Anna Bassols, Joaquim Segalés

**Affiliations:** 5 Centre de Recerca en Sanitat Animal (CReSA), UAB-IRTA, Campus de la Universitat Autònoma de Barcelona, Bellaterra (Cerdanyola del Vallès), 08193 Barcelona, Spain; 6 Departament de Producció Animal, ETSEA, Universitat de Lleida, 25198 Lleida, Spain; 7 Servei de Bioquímica Clínica Veterinària, Departament de Bioquímica i Biologia Molecular, Facultat de Veterinària, Universitat Autònoma de Barcelona, Bellaterra (Cerdanyola del Vallès), 08193 Barcelona, Spain; 8 Departament de Sanitat i Anatomia Animals, Universitat Autònoma de Barcelona, Bellaterra (Cerdanyola del Vallès), 08193 Barcelona, Spain; 9 Merial Laboratories, Barcelona, Spain

**Keywords:** Acute phase proteins, Average daily weight gain, Porcine circovirus type 2-systemic disease (PCV2-SD), PCV2 inactivated vaccine

## Abstract

The present work describes the serum haptoglobin (Hp) dynamics in piglets vaccinated and non-vaccinated with a commercial porcine circovirus type 2 (PCV2) vaccine at 3 weeks of age, and its relationship with the average daily weight gain (ADWG). The field study was carried out on two farms (A and B) with a previous clinical history of PCV2-systemic disease (PCV2-SD). The aim of the study was to assess whether Hp could be used as a surrogate marker of PCV2 vaccine efficacy. PCV2 infection was confirmed by quantitative real time PCR (qPCR) in pigs from both farms, but PCV2-SD was only diagnosed in farm A. No statistically significant relation was found between serum Hp concentration and the percentage of qPCR positive animals and the treatment applied (PCV2 vaccination) in both farms. On the other hand, using linear regression analysis, a significant negative correlation between the area under the curve of Hp (AUC_Hp_) and ADWG was observed for farm A (p < 0.00001) and B (p = 0.01). Based on the obtained determination coefficient (R^2^) values, AUC_Hp_ explained 20.0 and 11.6% of the observed ADWG for farms A and B, respectively. The present study supports that the measurement of acute phase proteins may be an indicator of ADWG in pig farms, but it was not apparently feasible to use the serum Hp concentration as a surrogate marker of PCV2 vaccine efficacy.

## Background

Porcine circovirus type 2 (PCV2)-systemic disease (PCV2-SD, formerly known as postweaning multisystemic wasting syndrome, PMWS), a disease primarily caused by PCV2, is considered one of the major swine diseases worldwide [1]. Traditionally, its control was based on counteracting the risk or triggering factors associated to this pathological condition, such as management improvement, control of co-infections and changes of the boar genetic background [2, 3]. However, commercial PCV2 vaccines available during the last 7 years have demonstrated to be very efficient to control PCV2-SD and PCV2 subclinical infection under natural conditions, as well as to improve production parameters such as average daily weight gain (ADWG) and mortality [4–8].

Acute phase proteins (APPs) are a group of blood proteins that change their concentration in animals subjected to external or internal challenges such as infection, inflammation, surgical trauma or stress [9, 10]. After the challenge, there is an increase in the plasma concentration of the so-called positive APPs, such as haptoglobin (Hp), Pig major acute-phase protein (Pig-MAP), serum amyloid A and C-reactive protein. On the other hand, other protein concentrations decrease, such as albumin, which represent the negative APPs. It has also been suggested that serum APPs may be used as an index for monitoring ADWG. In particular, low serum positive APPs have been correlated with better production parameters in pigs [11–13].

The aim of the present study was to describe the evolution of Hp serum levels in a population of pigs vaccinated and non-vaccinated against PCV2 and assess whether Hp could be used as a surrogate marker of PCV2 vaccine efficacy.

## Results and discussion

**Figure 1:**
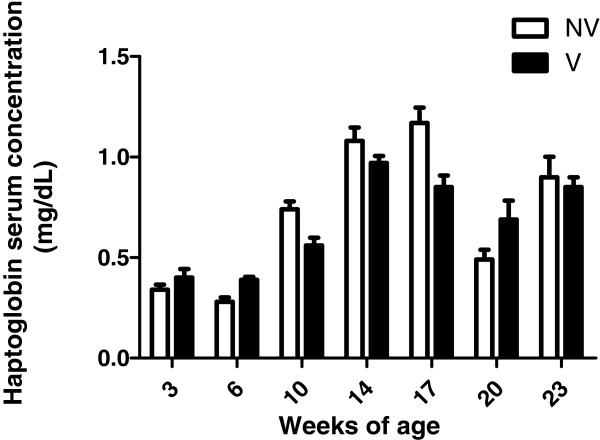
**Haptoglobin serum concentration (±SEM) at the different sampling times in fifty vaccinated (V) and fifty non-vaccinated (NV) pigs with a PCV2 vaccine at 3 weeks of age for farm A.** PCV2-SD was diagnosed around 12 weeks of age by means of clinical signs, histopathological findings and detection of PCV2 in tissues.

**Figure 2:**
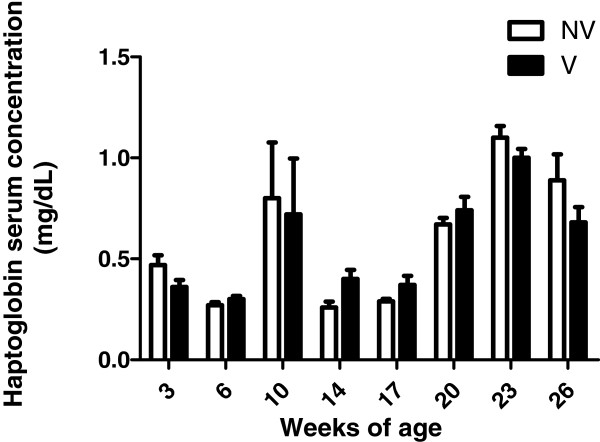
**Haptoglobin serum concentration (±SEM) at the different sampling times in fifty vaccinated (V) and fifty non-vaccinated (NV) pigs with a PCV2 vaccine at 3 weeks of age for farm B.** A clinical outbreak of colibacillosis and clinical signs of porcine respiratory disease complex were recorded at 10 and 23 weeks of age, respectively. No PCV2-SD was diagnosed in farm B.

Hp dynamics in serum samples from vaccinated and non-vaccinated pigs in farms A and B are shown in Figures 1 and 2, respectively.

Virological results of this study have been described in Fraile et al. [5]. Briefly, PCV2 in serum samples was absent (farm B) or with prevalence lower than 5% (farm A) until 14 weeks of age. Subsequently, both PCV2 prevalence and viral load increased progressively in both vaccinated and control groups, reaching a maximum prevalence at 17 and 20 weeks of age in farms A and B, respectively. In both farms, the percentage of qPCR positive pigs was significantly lower in vaccinated animals compared to non-vaccinated ones for several sampling periods throughout the trial [5]. Vaccinated animals in farm A showed lower mean serum Hp concentration than controls during the period of higher percentage of PCV2 qPCR positive animals (17 weeks of age, [5]). However, no statistically significant relation was found between serum Hp concentration and the percentage of qPCR positive animals and the treatment (PCV2 vaccination) in both farms. Moreover, no statistical relation was observed between AUC_Hp_ and AUC_PCV2_ for farm B but it was observed a statistical tendency (p = 0.07 and correlation coefficient of 0.19) for this relation in farm A. On the other hand, using linear regression analysis, a significant negative correlation between AUC_Hp_ and ADWG was observed for farms A (p < 0.00001) and B (p = 0.01). Based on the determination coefficient (R^2^) values obtained, AUC_Hp_ explained 20 and 11.6% of the observed ADWG for farms A and B, respectively. When data from both farms were analysed together, serum Hp concentration was significantly different taking into account farm and period of sampling as explanatory variables, whereas treatment effect was not significantly different, and only a statistical interaction (sampling time and treatment) was found for farm A.

It could be considered surprising that no significant differences were detected between the Hp serum concentration between vaccinated and non-vaccinated animals around PCV2 vaccination. The administration of a vaccine is considered an immunological systemic challenge for the animal, especially when administered intramuscularly. However, the specific product characteristics of the vaccine used indicate possible increase in rectal temperature up to 1.4°C (rarely higher than 2.5°C), lasting less than 24 hours [14]. Therefore, since the first blood sampling took place just before vaccination and the following one was 3 weeks post-vaccination, there were no real chances to detect an elevation of Hp due to vaccination.

Serum Hp concentration profiles were different depending on the farm tested in the present study. Clinical signs potentially compatible with PCV2-SD, characterized by growth retardation and increased mortality, were mainly observed around 2 months after vaccination in both farms (A and B), about 11–12 weeks of age. At that time it was observed a Hp serum concentration in pigs above a published reference value for this protein (0.9 mg/dL, [15]) in both farms, independently of the treatment applied. However, PCV2-SD was only confirmed by means of laboratory analyses in farm A [5]. According to the results, serum Hp concentration may be affected by PCV2 viremia in farms with a confirmed PCV2-SD diagnosis (farm A), mostly during the period of maximum prevalence of PCV2 infection but not in case of non-clinical/subclinical presentation (farm B). Since PCV2-SD is a chronic disease, usually associated with concurrent infections, it is very likely that the more persistent increased levels of Hp in farm A (Figure 1) would be related with this complex disease scenario. Importantly, an outbreak of colibacillosis and clinical signs of porcine respiratory disease complex were recorded at 10 and 23 weeks of age, respectively, in farm B. Accordingly, Hp serum concentration above 0.9 mg/dL (reference threshold in pigs) was also observed around those ages in this particular farm. Since the release pattern of this APP could vary in each farm due to the presence of different infectious and non-infectious factors, it was suggested that the two “peaks” of Hp observed in farm B (Figure 2), could apparently be associated to the presence of digestive and respiratory problems. As expected, these non-PCV2 associated clinical signs affected similarly to both PCV2 vaccinated and non-vaccinated animals.

The present study shows that the measurement of APPs may be an indicator of ADWG in pig farms. Specifically, lower Hp serum concentrations measured throughout the growing and fattening periods of the pigs (calculated as AUC_Hp_) were significantly correlated with improvement of production parameters (ADWG). These results are in agreement with published data that indicates a relationship between Hp serum levels and ADWG in pigs younger than 13 weeks old [12, 16], and also during the critical phase of post-weaning growth and adult pigs [11, 13]. Moreover, Hp was also found to be negatively correlated with growth rate in pigs fed a diet supplemented with β-glucans as growth promoter [17]. The APP response by itself may contribute to differences in ADWG since it has been suggested that, in diseased animals or even after vaccination, aminoacids may be diverted from muscle protein synthesis to APPs synthesis [18].

## Conclusions

It was not apparently feasible to use the serum Hp concentration pattern as a surrogate marker of PCV2 vaccine efficacy. This was probably due to the fact that an increase of Hp could be due to many factors not related with PCV2 infection dynamics or the effect of PCV2 vaccination. Obtained results fit with a previous work in which the increase of APP levels depended on the development of PCV2-SD at individual basis and not on infection with PCV2 at a subclinical level [19].

## Methods

Two farms (A and B), located in North-Eastern Spain, were selected based on their clinical history of PCV2-SD to evaluate the efficacy of an inactivated PCV2 vaccine licensed for piglets. Details of this field study have already been thoroughly described [5]. Briefly, from a total of 1,239 pigs, 619 of them received a single dose of 0.5 mL of CIRCOVAC^®^ at 3 weeks of age whereas 620 received the same amount of phosphate buffer solution (non-vaccinated animals). A subpopulation of 50 pigs from each group (vaccinated and non-vaccinated) in each farm were bled at 3, 6, 10, 14, 17, 20, 23 and 26 weeks of age. In the case of farm A, sampling was not carried out at 26 weeks of age because the bleeding at 23 weeks of age was close to the first shipment of pigs to the slaughterhouse. PCV2 load was investigated by means of a real-time quantitative PCR (qPCR) as previously described [20], and ADWG was also calculated for each individual pig [5].

Serum was obtained by centrifugation and frozen at -20°C until assaying. Hp was quantified by using a spectrophotometric method (hemoglobin binding assay) with commercial reagents from Tridelta Development Limited (Ireland), and performed on an automated analyzer (Olympus AU400, Hamburg, Germany). The intra-assay and inter-assay coefficient of variations were determined as previously described [21]. Detection limit was 0.05 mg/ml for Hp as indicated by the manufacturer.

All statistical analyses were carried out using the SAS system V.9.1.3 (SAS institute Inc, Cary, NC, USA). For all analyses, the individual pig was used as the experimental unit. The significance level (α) was set at 0.05. Area under the curve of Hp (AUC_Hp_) and the area under the curve for PCV2 viral load (AUC_PCV2_) versus time were calculated by the trapezoidal rule for each individual pig. A linear regression analysis was carried out to assess the strength of relation between AUC_Hp_ and AUC_PCV2_ for farms A and B. This same statistical tool was used to estimate the strength of relation between ADWG and the AUC_Hp_. Finally, GLM and MIXED model procedures of SAS system V.9.1.3 with repeated measures statement were used to analyze the effect of independent variables (X_i_): farm, PCV2 vaccination (treatment) and sampling period, and their interactions with Hp serum concentration (y dependent variable).

The experimental procedure was approved by the Ethical and Animal Welfare Committee of the *Universitat Autònoma of Barcelona* (Reference 665M2).

## Authors’ information

LF, SL, MN and JS are ECPHM diplomates.
